# Mechanisms of immune evasion in breast cancer

**DOI:** 10.1186/s12885-018-4441-3

**Published:** 2018-05-11

**Authors:** Joshua P. Bates, Roshanak Derakhshandeh, Laundette Jones, Tonya J. Webb

**Affiliations:** 1 Department of Microbiology and Immunology, University of Maryland School of Medicine and the Marlene and Stewart Greenebaum Comprehensive Cancer Center, 685 West Baltimore St; HSF I- Room 380, Baltimore, MD 21201 USA; 2 Department of Epidemiology and Public Health, University of Maryland School of Medicine and the Marlene and Stewart Greenebaum Comprehensive Cancer Center, Baltimore, MD 21201 USA

**Keywords:** Immunity, Immunotherapy, Lymphocytes, Cytokines, Dendritic cells, PD-1, Regulatory T cells, Myeloid derived suppressor cells

## Abstract

Tumors develop multiple mechanisms of immune evasion as they progress, with some cancer types being inherently better at ‘hiding’ than others. With an increased understanding of tumor immune surveillance, immunotherapy has emerged as a promising treatment strategy for breast cancer, despite historically being thought of as an immunologically silent neoplasm. Some types of cancer, such as melanoma, bladder, and renal cell carcinoma, have demonstrated a durable response to immunotherapeutic intervention, however, breast neoplasms have not shown the same efficacy. The causes of breast cancer’s immune silence derive from mechanisms that diminish immune recognition and others that promote strong immunosuppression. It is the mechanisms of immune evasion in breast cancers that are poorly defined. Thus, further characterization is critical for the development of better therapies. This brief review will seek to provide insight into the possible causes of weak immunogenicity and immune suppression mediated by breast cancers and highlight current immunotherapies being used to restore immune responses to breast cancer.

## Background

Cancer immune surveillance is an important process by which the immune system is able to monitor, recognize, and eliminate nascent tumor cells [1, 2]. There are three essential phases to this process termed elimination, equilibrium, and escape. Initially, innate and adaptive immune responses are able to control tumor growth. In this phase—elimination, acute inflammatory responses triggered by tumor-associated ‘danger signals’ initiates tumor cell recognition, the secretion of proinflammatory cytokines (notably, interleukin-12 (IL-12) and interferon-γ (IFN- γ), and killing by innate immune cells (e.g. natural killer (NK) cells, dendritic cells (DCs), and macrophages). Upon maturation, DCs migrate to nearby lymph nodes (LN), where they present tumor antigens and activate tumor-specific CD4^+^ and CD8^+^ T cells. These tumor-specific T cells will then migrate to the tumor site and facilitate killing. At this point, tumor cells are completely eradicated or resistant clonal variants develop. The clonal variants can develop resistance by decreasing their immunogenicity and/or secreting and recruiting immunosuppressive factors (several mechanisms of which are covered here). During this phase of equilibrium, if another cycle of immune responses is unable to eliminate the nascent cancer cells, then the phase of immune escape is reached, eventually leading to clinical manifestation. These phases together describe the theory of cancer immunoediting.

Ample evidence proves that neoplastic lesions are under immunosurveillance. Early proof of this was noted by pathologists who recognized that many patient tumors were densely infiltrated by innate and adaptive immune cells [3, 4]. Recent studies demonstrate that these immune cells are indeed mounting an antitumor response and that tumors develop mechanisms to combat an immune response [5, 6]. It has also been shown that mice lacking various components of the immune system have a greater risk of developing cancer than their immune competent counterparts [7–9]. A combined loss of lymphocytes by knockout of recombination activating gene-1 or − 2 (*rag*-1 or − 2), or by other methods, has demonstrated an even greater incidence of spontaneous and carcinogen-induced tumor formation in mice [2, 10]. Importantly, it has been demonstrated that cancer cells from immunocompromised mice are unable to initiate secondary tumors in syngeneic immunocompetent mice [2, 11]. In contrast, cancer cells isolated from immunocompetent mice are able to initiate tumors equally as well in both types of hosts (i.e. immune competent and incompetent) [4]. The leading explanation of this phenomenon is that the strongly immunogenic neoplastic cells developing in the immunocompetent host were eliminated by the immune system; however, the resistant variants give rise to a tumor that is more capable of evading immune destruction. However, tumors from immunocompromised mice have less selective pressures and are unable to evade immunosurveillance in immunocompetent mice and are therefore eliminated. Thus, immunocompetent mice develop stronger and more resistant tumors due to immunoediting [2]. It is possible that apoptosis of the strongly immunogenic clones is able to enhance antitumor immunity to the weakly immunogenic clones by mechanisms reviewed here. Thus, tumor cells and immune cells fight a silent battle, in which, after a phase of equilibrium and progression, cancer cells gain the upper hand and manifest macroscopically and clinically.

Why cancerous cells are able to escape immune surveillance is the question at hand. Clearly, the immune system’s surveillance of rogue cells plays a large part in the suppression of tumor escape, but for a variety of reasons cancers are still able to progress. Mechanisms of avoiding immune recognition include (and are most likely not limited to): low immunogenicity (e.g. tumor growth factor (TGF)-β, IL-10, indoleamine 2,3-dioxygenase (IDO) secretion), and extracellular matrix hindrance [12].

### BC tumor subtypes

Breast tumor subtypes are treated differently based on the status of molecular markers and associated class (i.e. basal-like, Luminal A, Luminal B, HER2-amplified). Standard therapy for estrogen receptor (ER) and/or progesterone receptor (PR)-positive tumors, which are also HER2 negative, are typically treated with hormonal therapy as a first line treatment and generally have a favorable prognosis compared to hormone receptor-negative tumors. Although there is no specific chemotherapeutic treatment regimen recommended by the American Society for Clinical Oncology for hormone-positive BC, other effective options include taxanes, anthracyclines, and platinum-based drugs. On the other hand, significant progress has been made in human epidermal growth factor receptor 2 (HER2/neu) overexpressing tumors within the past 5 years. The CLEOPATRA trial set the stage for establishing pertuzumab, an anti-HER2 dimerization inhibitor [13], in combination with trastuzumab plus chemotherapy as the standard for care in the adjuvant setting for HER2-positive BC; the regiment demonstrated a 6.1 month increase in overall survival (OS) [14]. Soon after, the NeoSphere and TRYPHAENA clinical trials would confirm pertuzumab’s safety and effectiveness in the neoadjuvant setting [15, 16]. Accordingly, results from the phase III APHINITY Trial, examining pertuzumab and trastuzumab plus chemotherapy in the adjuvant setting for operable, HER2-positive, primary BC, is highly anticipated with preliminary results reporting a positive outlook **(**NCT01358877). One of two antibody-drug conjugate treatments approved by the FDA, trastuzumab emtansine (T-DM1) (the other being brentuximab vedotin in Hodgkin’s Lymphoma) was successfully tested and shown to be more effective and less toxic than lapatinib plus chemotherapy, second line treatment for advanced HER2-postive BC in the EMELIA Trial [17]. Ongoing clinical trials and preclinical research should broaden T-DM1’s usefulness beyond advanced and/or metastatic disease given its low toxicity due to its specificity. Lapatinib, a tyrosine kinase inhibitor (TKI) of HER2, is also included in the standard secondary or tertiary treatment options for trastuzumab-resistant, advanced HER2-positive BC. In combination with chemotherapy, lapatinib effectively delays time to progression [18]. Multiple clinical trials are ongoing for more effective, and less toxic, TKIs; the most promising of which is neratinib, which recently “graduated” from the I-SPY 2 trial [19]. For patients with hormone-positive or triple-negative breast cancers (TNBC), targeted therapeutic options remain limited. Currently, TNBC or basal-like tumors (which is not a synonymous term, however both types show similar characteristics) are treated with a chemotherapeutic regimen including taxanes, anthracyclines, and/or cyclophosphamide. Clinicians generally agree that effective therapies for TNBC are lacking. Several very promising clinical trials involving targeted drug delivery and poly-ADP ribose polymerase (PARP) inhibition are currently underway.

While hormone-positive BCs have a relatively high 5 year OS with current, non-immunotherapeutic treatments, HER2 or TNBC subtypes of a similar stage have much poorer OS rates [20], and are more evasive, immunologically. However, the ability to predict disease progression and immunogenic potential is imperfect, and as such, researchers and clinicians are increasingly looking into gene expression profiling for molecular markers associated with immunogenicity to aid in characterization and treatment options. Determining the basis of BCs immunological evasiveness, as well as accounting for differences between patients, personalized immunotherapeutic methods should offer greater clinical benefit.

In recent years, much research has focused on the molecular reclassification of BC subtypes based on immunity-related genes (IRGs) in addition to the conventional intrinsic subtypes (Table 1). A gene expression profiling study by Staaf et al. found that in a panel of 58 HER2-amplified BCs, these tumors could be further subdivided into three subgroups with significant differences in prognostic outcome independent of stage, histological grade, or ER status. Importantly, one cluster had high invasive ability and a low immune response, and also correlated with the worst prognosis of the subtypes [21]. In an earlier study, ER-negative tumors could be subdivided into four main subtypes, with positive clinical outcomes associated with higher relative expression of complement and immune response pathway genes independent of lymphocytic infiltration [22]. Researchers have also demonstrated that TNBC can be subdivided into six molecular subgroups with unique gene expression profiles, and also were able to show differential responses to current chemotherapies in xenograft mouse models. Importantly, one of these subclasses was termed “immunomodulatory” [23] due to its signature expression of high levels of immune response (IR) genes. Additional studies evaluated the prognostic and predictive value of AR-positive TNBC and found that higher AR-positivity correlates with generally better clinical outcomes [24–28]. However, many studies have also correlated AR-positivity with a poor prognosis [29, 30]. Despite this ambiguity, these findings have quickly led to emerging therapies for TNBC targeted towards AR, including Enzalutamide and Bicalutamide (antiandrogens FDA-approved in metastatic castration-resistant prostate cancer), which are in early clinical trials (NCT02689427, NCT03055312, NCT00468715). Early results demonstrate a high prevalence of AR positivity and clinical benefit with only mild adverse events, an important factor when the first line treatment for TNBC, currently, is highly toxic. While it is relatively straightforward to develop targeted strategies for overexpression of primary drivers of malignancy (e.g. HER2, AR), doing so for “immunomodulatory” subtypes is often more complex and time-consuming. A meta-cohort of nearly 2000 tumor expression profiles demonstrated that certain subtypes of BC could be delineated by “metagene” classifiers specific to tumor-infiltrating immune cells, which also correlated with immune responsiveness quantified by immune pathway upregulation and differences in distant metastasis-free survival [31]. These researchers were able to extrapolate breast tumor phenotypes in to “immune benefit-enabled” and “immune benefit-disabled” while also predicting the ability of these subtypes to potentiate long-term, immune-mediated tumor rejection [31]. Currently, the biological attributes of the variety of BC subtypes may differ in their ability to sustain durable immune responses, however, recent data demonstrates varying levels of intratumoral immune cell-specific genes and immunogenic sensitivity, calling for future reclassification.

**Table 1:** Breast cancer subtype characterization and immune-related reclassification

Molecular Subtype	Gene expression pattern	Clinical features	Common treatment options	Potential reclassification
Luminal A	ER^+^ and/or PR^+^, HER2^−^, and low Ki67	30–70% prevalence; Tumor grade of 1 or 2	Endocrine therapy; Aromatase inhibitors; Standard chemotherapy; Best prognosis of the four tumor types	Further research required
Luminal B	ER^+^ and/or PR^+^ and HER2^+ or –^; High Ki67	10–20% prevalence; Often younger age of diagnosis; Higher grade and worse prognosis than luminal A	Endocrine therapy; Aromatase inhibitors; Standard chemotherapy	Further research required
HER-2-enriched	ER^−^, PR^−^, and HER2^+^	5–15% prevalence; Likely to be high grade and LN+; Poor prognosis	Trastuzumab; Pertuzumab; T-DM1; lapatinib; TKIs; anthracycline-based chemotherapy	SR +/−; CC +/−; IR +/−; ECM +/− (Teschendorff et al. [22]); (Staaf et al. [21])
Triple negative/basal-like	ER^−^, PR^−^, HER2^−^	15–20% prevalence; High grade and proliferation; Often BRCA-1 related;	Radiation; Platinum-based chemotherapy; PARP inhibitors	CC +/−; IR +/−; ECM +/− (Teschendorff et al. [22]); BL1, BL2, IM, MSL, LAR (Lehmann et al. [23])

*ER* estrogen receptor, *PR* progesterone receptor, *HER2* human epidermal growth factor receptor 2, *SR* steroid hormone response, *CC* cell cycle, *IR* immune response, *ECM* extracellular matrix, *BL1 and 2* Basal-like 1 and 2, *IM* immunomodulatory, *MSL* mesenchymal stem-like, *LAR* luminal androgen receptor, *LN* lymph node

Breast cancer is a heterogeneous neoplasm with many factors contributing to its intratumoral diversity, thus the various breast cancer subtypes offer different degrees of immunogenicity [32]. With the advent of more effective means of subtype characterization and stratification (specifically, genomic and transcriptomic analyses), in depth exploitation of immunomodulation and further characterization of biomarkers in BC can become more effective by researchers. Improving the stratification of BC subtypes with high throughput imaging and gene expression profiling, while also separating strongly immunogenic BC subtypes from the weakly immunogenic, will create more effective and personalized treatments and possibly explain why BC has been perceived as immunologically ‘silent’.

### Inflammation and breast cancer

In 1863, Rudolf Virchow proposed a functional relationship between inflammation and cancer. He hypothesized that the origin of cancer was at sites of chronic inflammation. It is now obvious that inflammatory cells have a potent impact on tumor development [33]. The pro-tumor actions of inflammatory cells include: the presence of leukocyte infiltration; the expression of cytokines such as tumor necrosis factor (TNF) or IL-1; chemokines such as CCL2 and CXCL8; active tissue remodeling and neo-angiogenesis. Tumor associated macrophages are important regulators in the link between inflammation and cancer [34, 35]. It took several years for researchers to prove that inflammation is fundamental to the growth and progression of breast cancer [36]. In 2009 a remarkable study confirmed the link between chronic inflammation and breast cancer recurrence [37]. The authors examined C-reactive protein (CRP) and serum amyloid A (SAA) levels, as measures of inflammation, and found that elevated CRP and SAA were associated with reduced disease-free survival in BC patients.

Most studies suggest that the inflammatory cells and cytokines found in tumors are more likely to contribute to immunosuppression, rather than induce effective antitumor responses [38–40]. Moreover, immune-compromised women exhibit reduced relative risk for common epithelial cancers, including breast adenocarcinoma [41, 42]. One previous study showed there was a 21% decrease in the risk of breast cancer among women who took NSAIDs at least twice a week for at least 5 years [43].

Although Virchow showed that cancer occurred at sites of chronic inflammation, Coley successfully treated sarcomas with bacterial mixtures, leading to tumor regression, mediated by acutely activated cytotoxic immune cells [44]. These paradoxical characteristics of leukocytes are due to functional plasticity of myeloid- and lymphoid-lineage cells. Macrophages, for example, when exposed to type 2 cytokines like IL-4, express epidermal growth factor (EGF) and vascular endothelial growth factor (VEGF), and enhance angiogenesis and mammary carcinoma metastasis. In contrast, macrophages activated through CD40 have antitumoral properties [45]. Several ongoing clinical trials target cytokines and growth factors for immune modulation, including cediranib, a VEGF inhibitor (see Tables 2-3).

**Table 2:** Ongoing immunotherapy/radiotherapy clinical trials

NCT Number	Phase	Regimen	Conditions	Enrollment
NCT02303366	I	Stereotactic ablation with anti-PD-1 antibody MK-3475	Oligometastatic breast cancer	15
T02730130	II	Pembrolizumab plus radiotherapy	Metastatic breast cancer	17
NCT02499367	II	Nivolumab after induction	Breast cancer	84
NCT02538471	II	LY2157299 Monohydrate and radiotherapy	Metastatic breast cancer	28
NCT01862900	I/II	Stereotactic body radiation with monoclonal antibody to OX40 (MEDI6469) after systemic therapy	Metastatic breast cancer	40
NCT01421017	I/II	Toll-like Receptor (TLR) 7 agonist, Cyclophosphamide, and radiotherapy	Metastatic breast cancer	55

### Breast microenvironment and lymphocytic infiltrate

BC cells themselves are master manipulators and evaders of immune destruction, and their mechanisms are not fully understood, fueling a stronger perception of BC’s poor immunogenicity. Determining their mechanisms of evasion is imperative for the development of more effective treatments. The most well characterized mechanisms outlining BC’s capacity to evade immune destruction are the expression of immune inhibitory co-stimulatory receptors (e.g. programmed cell death protein (PD)-1, cytotoxic T lymphocyte-associated protein (CTLA)-4, lymphocyte activation gene (LAG)-3, the presence of tumor-derived immunosuppressive factors (e.g. TGF-β, IL-10, IDO), and the infiltration of suppressive immune cells (e.g. regulatory T cells (Tregs), myeloid-derived suppressor cells (MDSCs), tumor-associated macrophages (TAMs) in the microenvironment. Moreover, it was shown that human BC cells can enhance self-tolerance by evading and altering the function of NK cells [46]. NK cells from non-invasive and invasive cancers were shown to decrease expression of activating receptors (such as NKp30 and NKG2D) and increase expression of inhibitory receptors (such as NKG2A), induced by multiple immunosuppressive cytokines (e.g. TGF-β and IDO) in the tumor microenvironment [46]. These many factors working in tandem demonstrate that the causes of BC’s weak immunogenicity are multifactorial.

Tumors that show greater immunogenicity and have greater infiltration of immune cells tend to be an indicator of response to chemotherapy and good prognosis, especially in TNBC and HER2-amplified BC [47–51]. BCs of any molecular subtype that contain greater than 50–60% lymphocytes in the tumor or stroma usually predict a relatively good prognostic outcome [52]. However, the tumor infiltrate composition can have conflicting and seemingly counterintuitive roles in creating a tumor-antagonizing or tumor-promoting environment [4]. This is another feature of BC that may be causing it to be thought of as an immunologically ‘silent’ neoplasm, although, recent studies have begun to shed light on the significance of TILs, and may potentially demonstrate immune cell-specific significance. Accordingly, specific TIL subtypes infer different prognostic value. In a recent meta-analysis, researchers found that high levels of PD-1^+^ TILs or FOXP3^+^ TILs predicts a poor prognosis, while higher levels of CD8^+^ TILs predicted a good prognosis [53]. Moreover, it was reported that TNBC patients with a high CD8/FOXP3 ratio in post-chemotherapeutically treated tumors had a better recurrence-free survival and breast cancer-specific survival [54]. High levels of CTLs alone [55], CD83^+^ DCs [56], CD20^+^ B cells [57], and, interestingly, CXCL13-producing CD4^+^ follicular helper T cells (Tfh) [58] have all be correlated with pathological complete response (pCR) in BC patients [52]. Importantly, many of these cell types are associated with the development of tertiary lymphoid structures—these structures represent foci of an ongoing adaptive immune response and may be linked to greater relapse free survival (RFS) and overall survival (OS) [59, 60]. Interestingly, Loi and colleagues recently demonstrated a strong link between the Ras-MAPK signaling pathway, PD-L1, and the abundance of TIL in the post-neoadjuvant setting of residual TNBC (which exhibit high rates of metastatic recurrence) [61]. They found that an increase in Ras-MAPK activation predicts a reduced TIL phenotype in the residual cancer, and to a lesser extent- with activation of cell-cycle pathways. Because Ras-MAPK activation is able to suppress inflammatory responses, such as secretion of IFN-γ and MHC expression, and increase PD-L1 and MEK activity, they hypothesized that MEK inhibition would reverse the phenotype. They went on to test the efficacy of combined MEK and PD-1/PD-L1 inhibition in vivo and in vitro*,* and found increased efficacy as indicated by tumor clearance.

These studies and other preclinical data highlight the importance of intratumoral lymphocytes, and led to the initiation of multiple clinical trials. The Eastern Cooperative Oncology Group (ECOG) conducted two-phase III trials (E2197 and E1199), with approximately 500 women treated within a 4-year period. The results from these studies confirmed stromal TILs as a robust and independent prognostic factor in TNBC; for every increase in lymphocytic infiltration, researchers found a concurrent decrease in risk of recurrence and death [50]. Intratumoral lymphocytes within HER2-amplified BCs have also been proven beneficial in early disease onset. The FinHER trial by Loi and colleagues reported a good prognosis for TNBC associated with TIL abundance confirming previous studies, although not in HER2-positive subtypes. However, they did find that increased TILs in HER2-amplified BC correlated with trastuzumab efficacy [49]. Thus, patients who have high relapse rates or do not find benefit from trastuzumab therapy, may be part of a low tumor infiltrate subset of patients, which calls for use of TIL as a predictive measure in treatment and, potentially, for the addition of checkpoint inhibitors to improve clinical outcomes. To confirm the significance of immunological modulation in the treatment of HER2-amplified BC and TNBC, the GeparSixto phase II clinical trial by Denkert et al. [48] evaluated immune-specific mRNA markers, such as immune-activating chemokines and immunosuppressive checkpoint molecules, in the tumors of 481 patients treated with neoadjuvant chemotherapy with or without carboplatin. Tumors with high lymphocytic infiltrate were found in 19.9% of HER2-amplified BC and 28.3% of TNBC and were an independent predictor of pCR [48]. Clearly, this balance between a tumor-promoting and tumor-antagonizing microenvironment is clinically significant and a promising therapeutic target for modulation.

### Standard treatments can induce antitumor immune responses

Chemotherapy-induced tumor cell death has been hypothesized in past years to engage antitumor immune response [62], and recent data show that conventional treatments, such as chemotherapy and radiotherapy, rely heavily on the immune response to be effective. Anthracyclines (such as doxorubicin) have been studied extensively for their ability to induce immunogenic cell death (ICD) [63, 64]. Anthracycline-based chemotherapy has been shown to induce a rapid translocation of calreticulin and heat shock proteins (HSPs) to the cell surface which stimulates the elimination of tumor cells by phagocytes, and the release of high mobility group box 1 (HMGB-1) —a ligand of toll-like receptor 4 (TLR4), triggering an innate anticancer immune response through the maturation of DCs [65–67].

Also included in the category of standard treatments for BC is trastuzumab—an anti-HER2 monoclonal antibody (mAb). HER2 is overexpressed in approximately 25% of BCs. While the majority of patients will initially respond to trastuzumab (65%), many will demonstrate disease progression within 12 months (52%) [68]. Mechanistic studies have shown that the treatment may rely on antibody-dependent cellular cytotoxicity (ADCC) primarily through NK cell activity [69]. Accordingly, in mice bearing HER2-overexpressing xenografts, 96% demonstrated tumor regression when treated with trastuzumab. In contrast, tumor-bearing mice lacking Fc receptor (FcR)-γ showed regression in only 29% [70]. Furthermore, in patients with HER2-amplified BC receiving trastuzumab plus taxane, or taxane alone for metastatic BC, abnormal FcR polymorphisms correlated with a decrease in progression free survival [71]. Due to the developed resistance seen in many HER2-positive BC patients, it will also be interesting to see how novel trastuzumab drug conjugates perform in the clinical setting [72]. These studies confirm the importance of ADCC for treatment with trastuzumab, and likely for other mAb-based therapies. As recently reviewed by Milani, the use of active immunotherapy (vaccines) in HER2-positive BC holds promise [73].

Combination chemotherapy has been shown to induce ICD and inhibit tumor-mediated immune suppression [74]. Tregs develop an increased frequency in association with BC progression, as well as with a biased towards a Th2 cytokine environment characterized by an increase in IL-4 and IL-10, and a decrease in IFN-γ and IL-2 in the plasma [75]. Importantly, pCR is associated with the disappearance of Tregs in breast carcinoma [76], validating the substantial immuno-suppressive capabilities of Tregs in the breast tumor microenvironment. It has been shown that the highly utilized chemotherapeutic, cyclophosphamide (CY), is able to induce cell death and inhibit the immunosuppressive capabilities of Tregs [77]. Moreover, a high CD8^+^/Treg tumor infiltrate ratio after neoadjuvant chemotherapy is a predictive factor of improved RFS and OS [52, 78]. CY was FDA approved as an anticancer agent in 1959 and this may explain its long-lived efficacy as a chemotherapeutic, and may have value in combination with immunotherapeutic treatments.

As mentioned above, anti-HER-2 monoclonal antibodies (mAb), i.e. trastuzumab, pertuzumab, and T-DM1 are included in the category of standard treatments for BC. HER-2 is overexpressed in approximately 25% of BC patients. While the majority of patients will initially respond to anti-HER2 therapy (65%), many will demonstrate disease progression within 8–18.5 months (52%) [14, 17, 68].

As ICD has emerged as one of the leading theories for reasons behind the effectiveness of conventional therapies, and defects in certain components of ICD (e.g. autocrine stimulation of type I IFN receptors [79], calreticulin cell surface expression [80], apoptotic release of adenosine triphosphate (ATP) and HMGB-1 [81]) have been implicated in the progression of cancer. Additionally, clinical studies examining the chemotherapeutic effect on BC found that leukocyte complexity and tumor-associated lymphocytes were independent predictors of response to chemotherapy and neoadjuvant chemotherapy [82].

Immunotherapy/radiotherapy combinations have promising potential to transform cancer treatment by harnessing the immune system in a synergistic approach. Increasing evidence demonstrates that radiation acts as an immune stimulus, recruiting immune mediators that enable anti-tumor responses within and outside the radiation field (known as the abscopal effect). The role of radiation is to diversify the T cell receptor repertoire of tumor infiltrating lymphocytes [83]. Combining radiotherapy with immunotherapy shifts the focus from direct tumor kill to immunomodulation, which is at least in part due to broadened neoantigen exposure, thus memory T cell repertoire expansion, T cell infiltration into tumor and enhanced T cell mediated tumor rejection [84]. The optimal dosing, fractionation, and target volume determination could be quite different from classic radiotherapy paradigms. Recently, scientists demonstrated the advantages of immunotherapy/radiotherapy in multiple tumor models, in metastatic solid tumors, particularly breast cancer and non–small-cell lung cancer (NSCLC) [85–87]. Table 2 provides a summary of ongoing clinical trials that combine immunotherapy with radiotherapy.

Importantly, the efficacy of radiotherapy and chemotherapy in mouse models of orthotopic BC increases with the depletion of immunosuppressive CD4^+^ T cells, macrophages, and Th2 cytokines [88].

### Applying immunotherapy to BC

The biggest obstacle facing BC immunotherapy is likely the conversion of non-immunogenic neoplasms to highly immunogenic and thus clinically responsive. Interestingly, in pancreatic ductal adenocarcinoma (PDAC), a purported ‘non-immunogenic’ neoplasm partially due to a complex microenvironment and low TILs, treated with irradiated, granulocyte-macrophage colony-stimulating factor (GM-CSF)-secreting, allogeneic PDAC vaccine (GVAX) in an adjuvant and neoadjuvant setting demonstrated the conversion of a non-immunogenic tumor to immunogenic. This conversion was confirmed by the development of tertiary lymphoid structures within the tumor microenvironment, an increase in TILs, expression of PD-1 and PD-L1, and prolonged overall survival [59]. Hopefully, treatments options and mechanisms for the immunogenic conversion seen in PDAC can be replicated in BC.

BRCA1/2 mutations are a well-known hereditary factor in BC. BRCA1/2 is crucial for providing genomic stability, while its loss is correlated with a high mutational load. Recent data suggests that high mutational burden may increase the variety of neoantigens available to induce an immune response, and therefore may be more responsive to immunotherapy [89–91]. This is especially true for pancreatic and ovarian cancers that are BRCA insufficient, and resultantly respond well to immunotherapy. Only recently, however, is this potential being exploited in BC with ongoing preclinical studies and several clinical trials underway (see Table 3). Alternatively, poly-(ADP-ribose) polymerase (PARP) inhibition in BRCA insufficient tumors takes advantage of the impaired DNA repair pathways (BRCA being responsible for homologous recombination, and PARP for base excision repair, primarily) allowing for exacerbation of DNA damage, ultimately leading to cell death [92, 93]. The first FDA approved PARP inhibitor, Olaparib, proved efficacious in relapsed and platinum-sensitive, BRCA mutated, ovarian cancers by significantly increasing PFS (8.4 months vs. 4.8 months) in the second phase, and reporting a substantial increase in Phase III of the SOLO-2 trial (NCT01874353) [94]. Of the subtypes of BC, TN is often BRCA mutated (30% [89];) and based on early results, may display the highest levels of mutational burden and neoantigen expression [95]. A similar phenotype in ovarian cancer warranted investigation into PARP inhibition, hopefully, BC may claim similar clinical benefit. Studies investigating PARP inhibition in combination with other therapies in TNBC are currently underway (see Table 3).

**Table 3:** Selected ongoing immunotherapy-based clinical trials

Patient population	Regimen	Phase	NIH No
HER2-negative advanced breast cancer	STEMVAC	I	NCT02157051
HER2-negative advanced breast cancer	WOKVAC	I	NCT02780401
HER2- negative metastatic breast cancer with BRCA1 or BRCA2 mutation	MEDI4736 with Olaparib	I/II	NCT02734004
HER2-negative metastatic breast cancer	MEDI4736 with Tremelimumab	II	NCT02536794
HER2-negative metastatic breast cancer	Pembrolizumab+Aromatase Inhibitor	II	NCT02648477
HER2-negative metastatic breast cancer	Pembrolizumab and Nab-paclitaxel	II	NCT02752685
Recurrent HER2-negative metastatic breast cancer	Opdivo & Abraxane	I	NCT02309177
Advanced triple negative breast cancer	Pembrolizumab plus chemotherapy	I/II	NCT02331251
Advanced triple negative breast cancer	AM0010 (recombinant human IL-10)	I	NCT02009449
Advanced triple negative breast cancer	MEDI4736 with Olaparib or Cediranib	I/II	NCT02484404
Advanced triple negative breast cancer	MEDI4736 with Vigil	II/III	NCT02725489
Advanced triple negative breast cancer	PVX-410 Vaccine in combination with Durvalumab	I	NCT02826434
Advanced triple negative breast cancer	Entinostat and Nivolumab with or without Ipilimumab	I	NCT02453620
Advanced triple negative breast cancer	Tremelimumab	II	NCT02527434
Advanced triple negative breast cancer	Atezolizumab+Nab-Paclitaxel	II	NCT02425891
Advanced triple negative breast cancer	PDR001	I/II	NCT02404441
Metastatic triple-negative breast cancer	Pembrolizumab plus chemotherapy	I/II	NCT02734290
Metastatic triple-negative breast cancer	Halaven & Pembrolizumab	I/II	NCT02513472
Metastatic triple-negative breast cancer	Pembrolizumab with Carboplatin and Gemcitabine	II	NCT02755272
Metastatic triple-negative breast cancer	Pembrolizumab plus radiotherapy	II	NCT02730130
Metastatic triple-negative breast cancer	TAK-659 with Nivolumab	I	NCT02834247
Metastatic triple-negative breast cancer	CSF1R Inhibitor (PLX3397) with Pembrolizumab	I/II	NCT02452424
Metastatic triple-negative breast cancer	Single-dose Cyclophosphamide +Pembrolizumab	II	NCT02768701
Metastatic triple-negative breast cancer	Pembrolizumab	I	NCT02447003
Metastatic triple-negative breast cancer	Pembrolizumab	III	NCT02555657
Metastatic triple-negative breast cancer	Niraparib with Pembrolizumab	I/II	NCT02657889
Stage I-III triple negative breast cancer	MEDI4736 and chemotherapy before surgery	I/II	NCT02489448
HER2+ breast cancer	NeuVax with Herceptin	II	NCT01570036
HER2+ breast cancer	Atezolizumab with Trastuzumab Emtansine or with Trastuzumab and Pertuzumab	I	NCT02605915
HER2+ advanced breast cancer	AdHER2/neu dendritic cell vaccine	I	NCT01730118
ER+, stage I, II or III breast cancer	MONTANIDE™ ISA 51 VG combined with neoadjuvant chemotherapy	I/II	NCT02229084
Metastatic breast cancer	Hypofractionated radiotherapy with MEDI4736 and Tremelimumab	I	NCT02639026
Persistent Triple-Negative Disease	Personalized polyepitope DNA vaccine following neoadjuvant chemotherapy	I	NCT02348320

Note: STEMVAC, WOKVAC, Vigil, NeuVax, MONTANIDE™ ISA 51 VG (vaccines); MEDI4736, Atezolizumab (anti-PD-L1), Olaparib, Niraparib (PARP inhibitor); Tremelimumab, Ipilimumab (anti-CTLA-4); Pembrolizumab; Nivolumab, PDR001 (anti-PD-1); Entinostat (HDACi); TAK-659 (SYKi). Data extracted from https://www.breastcancertrials.org

**Fig. 1:**
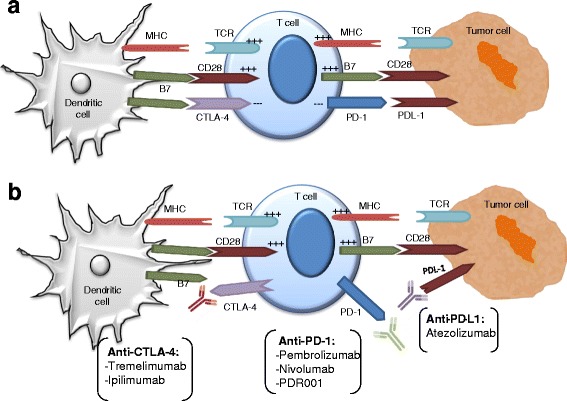
Restoring T-cell activation through the use of checkpoint inhibitors. **a** Naïve T cells become activated following their recognition of peptides presented in the context of MHC molecules expressed on the surface of antigen presenting cells, such as dendritic cells, along with engagement of costimulatory molecules (B7) with CD28 and this activation results in upregulation of cytotoxic T-lymphocyte antigen 4 (CTLA-4). The CTLA-4 receptor on T lymphocytes is a negative regulator of T cell activation that outcompetes CD28 for binding to B7 on antigen presenting cells in order to block T cell responses. Another inhibitory pathway uses the programmed cell death 1 (PD-1) receptor. CTLA-4 and PD-1 modulate different aspects of the T cell response. CTLA-4 is rapidly induced in T cells, following activation via MHC/TCR and B7/CD28 mediated signaling. In contrast, the major role of the PD1 pathway is to regulate inflammatory responses in tissues by effector T cells recognizing antigen in peripheral tissues. **b** Cancers can express the ligands for these checkpoint molecules, thus blocking T cell responses. Thus, the use of checkpoint inhibitors allow T cells to maintain their effector functions via the secretion of cytokines that recruit other immune cells to participate in the antitumor response and through their cytolytic capabilities. Numerous checkpoint inhibitors are currently being used in the clinic. CTLA-4, cytotoxic T-lymphocyte antigen; PD-1, programmed death 1; PD-L1, programmed death ligand 1; APC, antigen presenting cell; MHC, major histocompatibility complex; TCR, T cell receptor

Inhibitory receptors such as PD­1 and CTLA-­4 expressed on tumor ­specific T cells lead to suppression of effector functions such as proliferation, cytokine secretion, and tumor cell lysis [96–98] (see schematic in Fig. 1). PD-L1 expression has been observed in melanoma, lung, breast, ovarian, esophageal, pancreatic, bladder, kidney, and hematopoietic malignancies [99]. Immunologic checkpoint blockade with monoclonal antibodies that target CTLA-4 (ipilimumab) and PD-1/PD-L1 (nivolumab/pembrolizumab) have proven to be effective for the treatment of multiple malignancies. Ipilimumab is the first agent that demonstrated improved OS in phase III trials of melanoma patients. Anti-PD­1 antibody and one of its ligands, PD­L1, have shown much promise in the treatment of melanoma, renal cell cancer, non­small cell lung cancer, and other tumors [96].

Previous studies have shown that PD-L1 is expressed in approximately 20% of TNBC cases. Importantly, increased PD-L1 expression on the surface of TNBC cells led to decreased T cell proliferation and increased apoptosis [100]. These observations provide the rationale for implementing therapeutic strategies targeting the PD-1/PD-L1 axis in TNBC. An early phase I clinical trial of 26 patients with advanced, hormone-responsive BC, tremelimumab (anti-CTLA-4 mAb) used in combination with exemestane, an aromatase inhibitor, demonstrated an overall response rate of stable disease for more than 12 weeks with mild treatment-related adverse events [101]. Furthermore, a study examined the expression of CTLA-4 in human BC and found that a high density of interstitial CTLA-4^+^ lymphocytes correlated with increased DFS and OS, in contrast highly expressing CTLA-4^+^ tumors were correlated with a shorter DFS and OS. Thus, patients with high CTLA-4^+^ lymphocytes and CTLA-4^low^ tumors had the best prognosis, and these results may be important for determining patients who would benefit most from anti-CTLA-4 mAb therapy [102]. Many promising studies have shown efficacy in combination therapy, so it will be interesting to see if synergistic combinations will also show efficacy towards the low immunogenicity of BC. Current clinical trials attempting to modulate immunity and attain durable responses in BC via PD-1 blockade along with standard treatment options, include TONIC phase II trial (NCT02499367) for the treatment of TNBC, and the phase Ib/II clinical trial PANACEA (NCT02129556), which is studying the efficacy in trastuzumab-resistant HER2-amplified BC. Other clinical trials are highlighted in Table 3.

Immune checkpoint blockade therapy in BC has shown promise. MEDI4736, an anti-PD-L1 checkpoint inhibitor made by MedImmune/AstraZeneca, is being tested in three trials: a phase I trial of MEDI4736 for patients with solid tumors, including breast cancer (NCT01693562); a phase I trial of MEDI0680 (AMP-514), an anti-PD-1 antibody, and MEDI4736 in patients with advanced cancers (NCT02118337); and a phase I/II trial of MEDI6469, an anti-OX40 agonist antibody, alone or with tremelimumab, an anti-CTLA-4 antibody, and/or MEDI4736 (NCT02205333).

### Adoptive cell immunotherapy

**Fig. 2:**
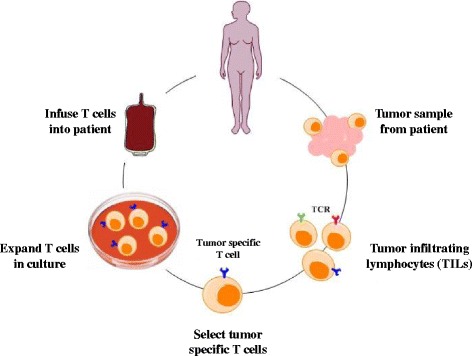
Adoptive T cell immunotherapy. Tumor mass can be surgically excised, fragmented, and placed in a flask, which contains T cell growth factors, such as interleukin-2 (IL-2). This will induce the proliferation of tumor-infiltrating lymphocytes, in order to expand tumor-specific T cells. Expanded tumor specific T cells will be reinfused into cancer patients in order to induce potent anti-tumor immune responses

Immunotherapy has been long lauded as a potentially powerful breast cancer treatment, one that can be more effective than the conventional therapies of surgery, radiation or chemotherapy. Perhaps even more promising in BC immunotherapy, is the development of adoptive cell and vaccine-based therapies. Initial approaches to adoptive cellular immunotherapy involved purifying TILs from metastatic foci, expanding them ex vivo in the presence of high-dose IL-2, and then infusing them back to the patient. Effectiveness of these therapies will depend on their ability to target potent tumor-specific or tumor-associated antigens, overcome the mechanisms of immune tolerance, and nullify immunosuppressive pathways (e.g. PD-1/L1, CTLA-4, etc.) [103]. A meta-analysis of data from 633 BC patients sought to evaluate the therapeutic efficacy of adoptively transferred autologous DCs, cytokine-induced killer (CIK) cells, or DC-CIK in combination. Results found only mild adverse events across studies and that combination treatment significantly improved 1-year survival, which correlated with increased production of IL-2, IL-6, IFN-γ, and TNF-α in the peripheral blood [104]. Although this approach is more complex and expensive, adoptive cellular immunotherapy shows great potential in the clinical setting (Fig. 2 & Table 4).

**Table 4:** Ongoing clinical trials using adoptive cell therapy in breast cancer patients

Patient population	Intervention	Phase	Country	NIH No
Metastatic breast cancer refractory to at least 1 standard therapy	cMet CAR RNA T Cells Targeting Breast Cancer	I	USA	NCT01837602
Malignant pleural disease, Mesothelioma, Lung Cancer, Breast Cancer	Autologous T Cells Genetically Engineered to Target the Cancer-Cell Surface Antigen Mesothelin	I	USA	NCT02414269
Solid tumors	Tumor Associated Antigen (TAA)-Specific Cytotoxic T-Lymphocytes	I	USA	NCT02239861

## Conclusions

Considerable clinical and preclinical evidence shows that BC is under immunosurveillance and we are just beginning to understand the complex interplay of the immune system and BC. It is important to understand the complexity of immunology and that no singular therapy will likely be the most effective treatment. The challenge researchers currently face is determining strategies and methods to modulate an effective immune response against BC.

Lymphocytic infiltrate has proven to be a strong prognostic indicator of pCR and OS in several types of cancers. Thus, it will be interesting to see how BCs can be further subdivided into TIL^+^, TIL^−^, or even TIL-intermediate variations, and what implications those variations might have [52]. Moreover, it might be necessary to further subdivide the TIL status of breast neoplasms to the individual cell types. Clinical relevance of TILs drives us to research novel methods that can be used to integrate immunotherapy with conventional therapy [105]. It will be interesting to see if the study by Loi et al. [61] demonstrating the link between MEK and PD-L1 expression will attain clinical interest; the findings may not just be specific to BC and could have wide ranging benefits across multiple disease types.

Current chemotherapeutic and radiotherapeutics seem to be particularly effective if they elicit a robust immune response. Therefore, conventional treatments combined synergistically with immunotherapy or combination immunotherapy should increase their efficacy. For example, CTLA-4 blockade combined with local radiation inhibits lung metastasis in a mouse model of BC [85]. The use of anti-PD-1 mAb in combination with a multi-peptide vaccine prolonged survival in tumor-bearing mice [106]. One study also demonstrated the importance of careful scheduling for efficient immunotherapy in a mouse model of BC by showing that concurrent delivery of a protein tyrosine kinase inhibitor with a vaccine inhibits an immune response, while sequential delivery allows for more effective priming of the immune response to the vaccine [107]. Additional studies are needed to determine effective regimens, those that promote the most synergy, while also accounting for scheduling and toxicities. Immunotherapeutic strategies in BC and their efficacy for the treatment of specific BC subtypes are only in their early stages. The advent of better methods of cancer cell characterization, identification of definitive biomarkers, and the development of rationally designed immunotherapeutic approaches will undoubtedly lead to improved survival and an increase in the overall quality of life in breast cancer patients.

## Abbreviations

ADCC: Antibody-dependent cellular cytotoxicity
ATP: Adenosine triphosphate
BC: Breast cancer
CIK: Cytokine-induced killer
CTL: Cytotoxic T lymphocytes
CTLA-4: Cytotoxic T lymphocyte associated protein 4
CY: Cyclophosphamide
DC: Dendritic cell
EGF: Epidermal growth factor
ER: Estrogen receptor
FcR: Fc receptor
GM-CSF: Granulocyte macrophage-colony stimulating factor
HER2: Human epidermal growth factor receptor 2
HMGB-1: High mobility group box 1
HSPs: Heat shock proteins
ICD: Immunogenic cell death
IDO: Indoleamine 2,3-dioxygenase
IFN: Interferon
IL: Interleukin
IRG: Immunity-related genes
LN: Lymph node
mAb: monoclonal antibody
MDSC: Myeloid-derived suppressor cell
NK: Natural killer
OS: Overall survival
pCR: Pathological complete response
PD-1: Programmed cell death protein 1
PDAC: Pancreatic ductal adenocarcinoma
PD-L1: Programmed death-ligand 1
PR: Progesterone receptor
RAG: Recombination-activating gene
RFS: Relapse free survival
TAM: Tumor-associated macrophages
Tfh: T follicular helper
Th: Helper T cell cells
TIL: Tumor-infiltrating lymphocytes
TKI: Tyrosine kinase inhibitor
TLR: Toll-like receptor
TNBC: Triple negative breast cancer
TNF: Tumor necrosis factor
Treg: Regulatory T cells
VEGF: Vascular endothelial growth factor
